# Small terrestrial mammals (Rodentia and Soricomorpha) along a gradient of forest anthropisation (reserves, managed forests, urban parks) in France

**DOI:** 10.3897/BDJ.10.e95214

**Published:** 2022-12-30

**Authors:** Julien Pradel, Marie Bouilloud, Anne Loiseau, Sylvain Piry, Maxime Galan, Emmanuelle Artige, Guillaume Castel, Julien Ferrero, Romain Gallet, Geoffrey Thuel, Nathalie Vieira, Nathalie Charbonnel

**Affiliations:** 1 CBGP, INRAE, CIRAD, Institut Agro, IRD, University of Montpellier, Montpellier, France CBGP, INRAE, CIRAD, Institut Agro, IRD, University of Montpellier Montpellier France; 2 MIVEGEC, IRD, CNRS, University of Montpellier, Montpellier, France MIVEGEC, IRD, CNRS, University of Montpellier Montpellier France

**Keywords:** rodents, shrews, community, biodiversity, forests, urban parks, global changes, dilution effect

## Abstract

**Background:**

Understanding the relationships between wildlife biodiversity and zoonotic infectious diseases in a changing climate is a challenging issue that scientists must address to support further policy actions. We aim at tackling this challenge by focusing on small mammal-borne diseases in temperate forests and large urban green spaces. Small mammals are important reservoirs of zoonotic agents, with a high transmission potential for humans and domestic animals. Forests and large urban green spaces are ecosystems where efforts are undertaken to preserve biodiversity. They are put forward for their contribution to human well-being in addition to other ecosystem services (e.g. provisioning and regulating services). Moreover, forests and large urban green spaces are environments where small mammals are abundant and human/domestic-wildlife interactions are plausible to occur. These environments are, therefore, focal points for conservation management and public health issues.

**New information:**

The European Biodiversa BioRodDis project (https://www6.inrae.fr/biodiversa-bioroddis) aims at better understanding the relationships between small terrestrial mammal biodiversity and health in the context of global change and, in particular, of forest anthropisation and urbanisation. Here, we present the data gathered in France. The dataset will enable us to describe the diversity of small terrestrial mammal communities in forested areas corresponding to different levels of anthropisation and to evaluate the variability of this diversity over time, between seasons and years.

The dataset contains occurrences of small terrestrial mammals (Rodentia and Soricomorpha) trapped in forested areas in eastern France (administrative Departments: Rhône, Ain, Jura). The sampling sites correspond to different degrees of anthropisation. Forests included in biological reserves are the least anthropised sites. Then, public forests and urban parks experience increasing levels of anthropisation. Data were collected during spring and autumn 2020 (three to four sampling sites), 2021 (six sampling sites) and 2022 (four sampling sites). These variations in the number of sites between years were due to lockdown restrictions in 2020 or to the legal authorisation to trap around biological reserves granted in 2021 only. The capture of animals was carried out in various types of forests (pine, deciduous, mixed) and in different habitats within urban parks (wooded areas, buildings, hay storage yards, riverside vegetation, restaurants, playground for kids, botanical garden, landfills). Animals were captured using live traps that were set on the ground for one to 11 nights. During this study period, 1593 small mammals were trapped and identified. They belong to 15 species, amongst which were nine species of rodents (Muridae, Cricetidae, Gliridae) and six species of shrews (Soricidae). They were weighted (gram) and measured (cm): head-body length, tail length and hind-foot length. Sexual characteristics were also recorded.

## Introduction

These last centuries, humans have strongly impacted ecosystems, through the development of activities including, amongst others, land-use changes, overexploitation of natural resources including wood or introduction of non-native species (Ellis et al. 2013). Urbanisation, the process of environmental change resulting from dense human presence and occupancy, is an extreme case of anthropogenic transformation.

Anthropisation and, in particular, urbanisation, have led to unprecedented levels of disturbance, disrupting ecological processes, eroding biodiversity and modifying communities towards simplified assemblages (Grimm et al. 2008). Indeed, not all species may adapt to such rapid and intense changes associated with anthropisation. This may result in an impoverishment in the diversity of communities and the selection of species characterised by particular life history traits that enable them to tolerate or benefit from these new environmental conditions (Santini et al. 2018). These shifts in species diversity and composition may have pervasive cascading effects on public health.

First, biological diversity alteration may be associated with the disruption of the regulation of pathogen circulation or emergence, including zoonotic ones. Two processes may interact to influence disease risk in anthropised ecosystems. On the one hand, high host diversity can “dilute” pathogen transmission. Such “dilution effect” occurs when the diversity of an ecological community reduces the transmission of a pathogen. It may occur when species vary in their competence (i.e. their ability to harbour and transmit a specific pathogen) and when species diversity limits encounter rates and enhances host regulation (Keesing et al. 2006). As such, negative anthropogenic impacts on diversity may prevent dilution effects and increase pathogen transmission. On the other hand, biodiversity may be associated with enhanced risk of pathogen emergence. This “amplification effect” occurs when higher diversity leads to higher host density, contact rates and/or transmissibility (Keesing et al. 2006). Moreover, each reservoir species may bring its own pathogens, in particular, non-native species can lead to the introduction of new pathogens. Second, animal life history traits associated with adaptation to human pressures (e.g. small body size, fast life history) also seem to correlate with reservoir competence (Gibb et al. 2020, Albery et al. 2021).

As such, it is urgent to elucidate the interlinkages between anthropisation and animal community diversity and assemblages, to better predict and prevent zoonotic diseases risk. Several studies have described biodiversity changes for animal communities along anthropisation gradients (e.g. Cavia et al. 2009, Matthies et al. 2017). Nevertheless, there is little information on the effect of anthropisation on mammal communities, most studies focusing on birds or insects.

We provide occurrence data on small mammals (rodents and shrews) surveyed during two years along a gradient of forest anthropisation, including biological reserves, managed forests and urban parks, in eastern France. Small mammals are important reservoirs of zoonotic agents; forests and urban green spaces are environments where small mammals are abundant, human/domestic-wildlife interactions are plausible to occur and efforts are undertaken to preserve biodiversity, while limiting disease risk. These data will hence contribute to advancement of the knowledge of:

1. the distribution of small mammals in this geographic area, including urban parks that have been scarcely studied and
2. the shifts in community diversity and assemblage associated with anthropisation.

## General description

### Purpose

This paper provides data collected during the Biodiversa Bioroddis project in France (2020-2022). The dataset contains occurrences of small terrestrial mammals (Rodentia and Soricomorpha) trapped in forested areas in eastern France (administrative Departments: Rhône, Ain, Jura). The sampling sites correspond to different degrees of anthropisation. Forests included in biological reserves are the least anthropised sites. Public forests and urban parks experience increasing levels of anthropisation. The dataset will enable us to describe the diversity of small terrestrial mammal communities in forested areas corresponding to different levels of anthropisation and to evaluate the variability of this diversity over time, between seasons and years.

## Project description

### Title

BioRodDis: Managing BIOdiversity in forests and urban green spaces - Dilution and amplification effects on RODent microbiomes and rodent-borne DISeases

### Personnel

Coordinator: Charbonnel Nathalie

### Study area description

BioRodDis includes occurrence of small terrestrial mammals from forests and urban parks in five countries: Belgium, France, Germany, Ireland and Poland.

### Design description

The BioRodDis project aims at elucidating the interlinkages between small mammal biodiversity and diseases at local and European scales using standardised assessments of biodiversity and disease risks. In particular, the dilution/amplification effect is assessed by integrating new key research directions, i.e. host microbiome and multiple pathogen diversity levels on one hand, seasonal and multi-annual dynamics on the other hand, including climate change scenarios and interactions with socioeconomic contexts. More information is provided in the website: https://www6.inrae.fr/biodiversa-bioroddis.

### Funding

This project is funded through the 2018-2019 BiodivERsA joint call for research proposals, under the BiodivERsA3 ERA-Net COFUND programme, and with the funding organisations ANR (France), DFG (Germany), EPA (Ireland), FWO (Belgium) and NCN (Poland).

## Sampling methods

### Study extent

**Figure 1.**
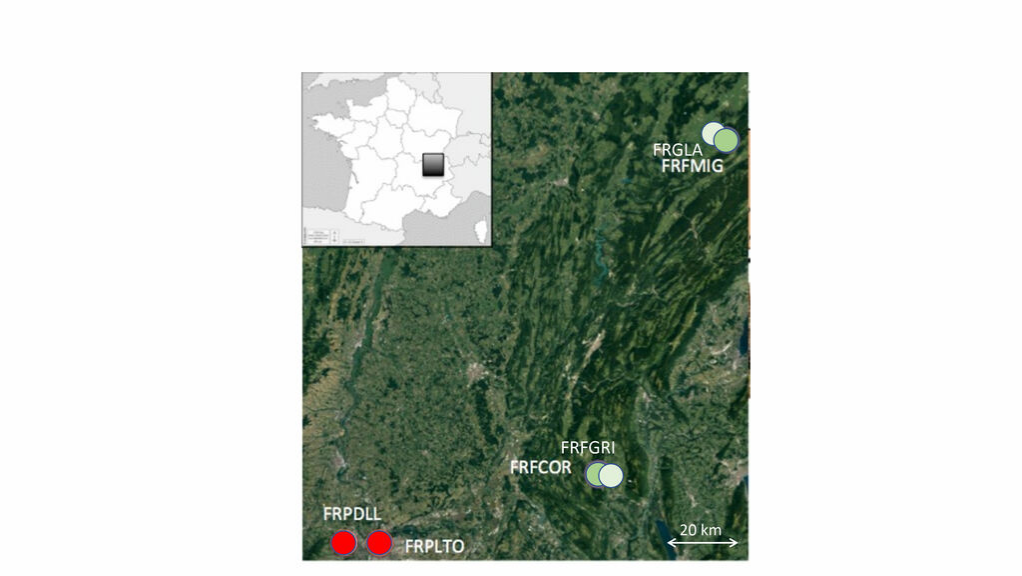
General map of the studied areas and their position in France. Localities are indicated by circles, red circles = urban parks; green circles = managed forests; light green circles = protected forests. FRPLTO: Lyon, Parc de la Tête d’Or; FRPDLL: Marcy l'étoile, Domaine Lacroix Laval; FRFCOR: Cormaranche en Bugey; FRFGRI: Arvière, La Griffe au diable; FRFMIG: Mignovillard; FRFGLA: Esserval-Tartre, La Glacière.

**Figure 2.**
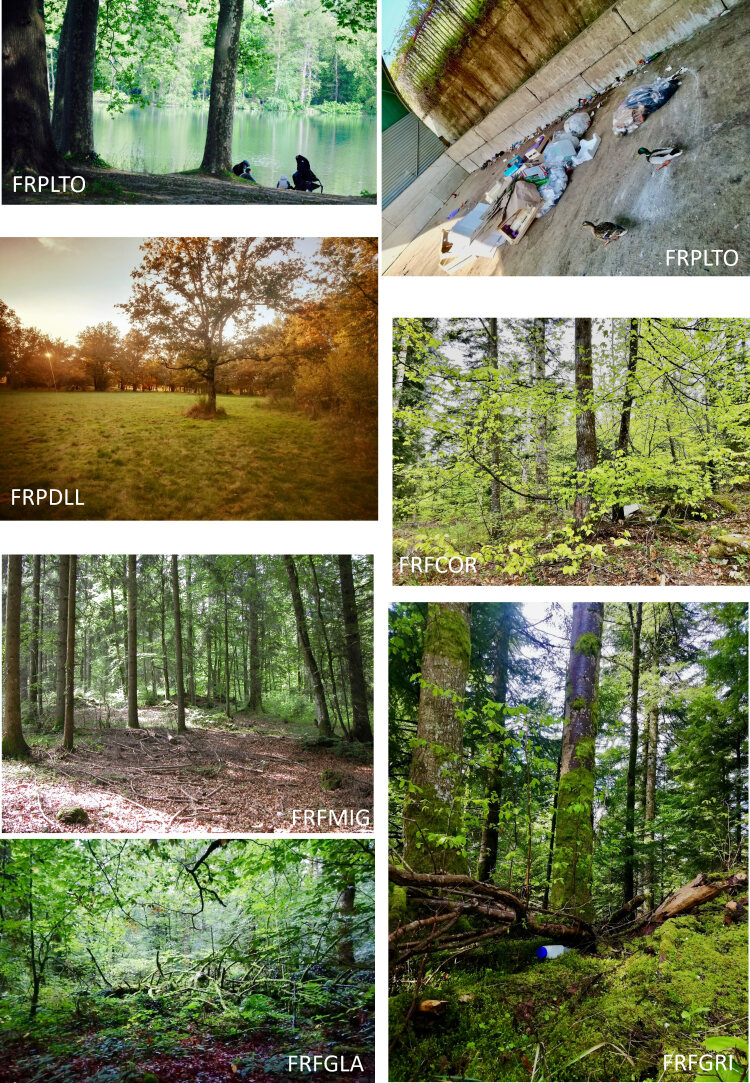
Pictures of some of the trapping lines within each locality. FRPLTO: Lyon, Parc de la Tête d’Or; FRPDLL: Marcy l'étoile, Domaine Lacroix Laval; FRFCOR: Cormaranche en Bugey; FRFGRI: Arvière, La Griffe au diable; FRFMIG: Mignovillard; FRFGLA: Esserval-Tartre, La Glacière

This study includes six sampling sites (2 forest reserves, 2 public forests, 2 urban parks) in eastern France (see Figs 1, 2). The current dataset extends from February 2020 to June 2022.

### Sampling description

Small mammals were live-trapped using INRA traps for all rodents and shrews, except rats (an INRA trap is composed of a 160 × 50 × 50-mm aluminium tunnel, coupled with a 150 × 70 × 70-mm plastic rest box). Rats were trapped using meshed traps of 500 x 175 x 175 cm (Fig. 4).

**Figure 3.**
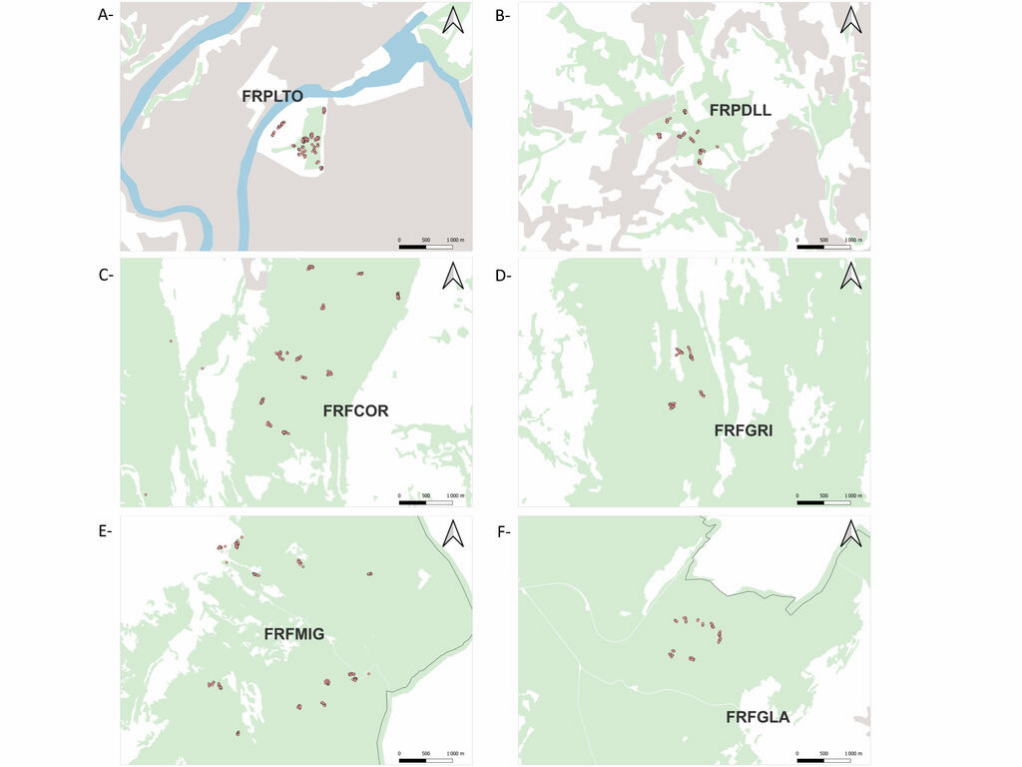
Maps of the trapping lines (red points) represented at a local scale for each of the following localities: A- FRPLTO: Lyon, Parc de la Tête d’Or; B- FRPDLL: Marcy l'étoile, Domaine Lacroix Laval; C- FRFCOR: Cormaranche en Bugey; D- FRFGRI: Arvière, La Griffe au diable; E- FRFMIG: Mignovillard; F- FRFGLA: Esserval-Tartre, La Glacière. The landscape around the trapping lines is represented by different colours corresponding to water bodies (blue; corine land cover 5), Forest and semi-natural areas (green, Forest database), artificial areas (grey; corine land cover 1) or other land-cover classes (white).

Six to ten lines of 20 INRA live-traps and one meshed trap, with about 3 m interval, were set up so that each sampling locality consisted of a few km^2^ area (see Table 1, Fig. 3). The rest box was filled with hydrophobic cotton to provide good conditions for trapped animals. All traps were baited with sunflower seeds, carrots and sardine. Each trap was geolocated. The traps were checked daily, early in the morning. Trapping session per locality lasted at least three nights, except when abundances were too low and new trap lines had to be set up. Moreover, because we encountered difficulties in trapping rats and mice in urban parks, traps were set in particular places where animals had been detected and they were removed after 10 to 11 nights (see details in Suppl. material 1). For ethical reasons, we could not trap more than 35 animals per species, locality and session. As such, these were released individuals when we reached 35 individuals of a species for a given locality and session. Relative abundance (e.g. trapping success) might nevertheless be estimated using all capture information gathered from the three first trapping nights (see details in Suppl. material 1). In addition, note that rare shrews were released for all sessions, except autumn 2020 and spring 2021, to limit our impact on these populations. Altogether, this dataset gathered information on 1593 small mammals and 21,494 trap-nights. The details of the number of animals dissected per locality and session are provided in Table 1.

### Quality control

All captured animals were determined to species level using morphological criteria in the field or using molecular methods when necessary (CO1 sequencing for *Microtus* species and shrews, Pagès et al. 2010 or DNA fingerprinting for *Apodemus* species, Bugarski-Stanojević et al. 2013, Suppl. material 2). Animal dissections and measurements were performed according to the protocols described in Herbreteau et al. (2011). Biological samples (organs, blood, parasites) were deposited in a public biobank at the Centre de Biologie pour la Gestion des Populations (CBGP) in Montpellier, France. Metadata associated with the samples and occurrence set were deposited in the CBGP small mammal database (BPM, Base Petits Mammifères, https://doi.org/10.15454/WWNUPO).

### Step description

**Figure 4.**
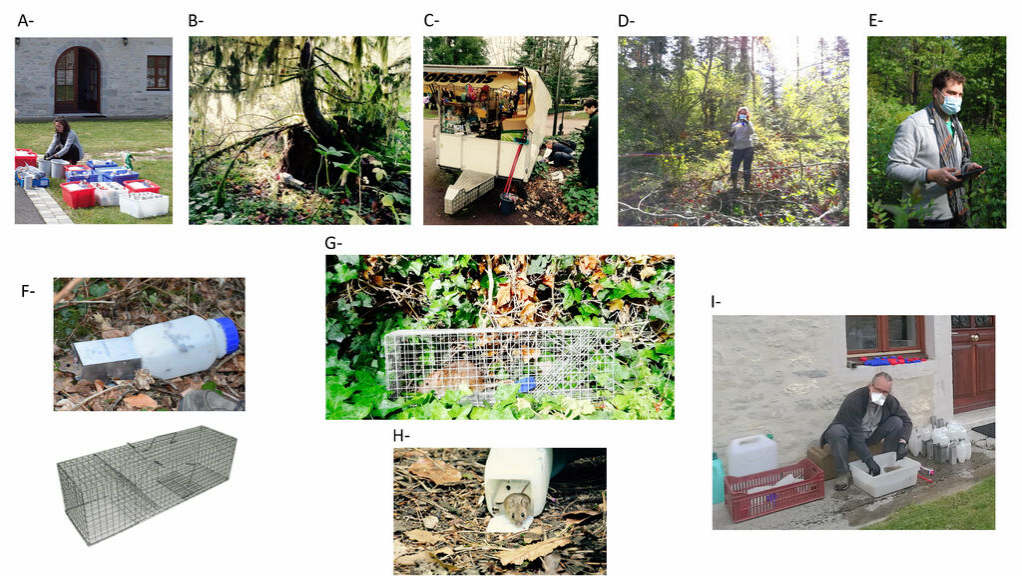
Pictures illustrating the different steps leading to trapping and individual information. A- Preparation of traps (bait, cotton, trap number); B- An INRA trap sets in a forest; C- Meshed trap sets in a urban park; D- Trap checking in the morning (masks and gloves are important to protect animals and humans from zoonotic agents); E- Capture information recorded on a digital tablet (this picture was taken during the lockdown, which explains the mask); F- An INRA trap used to capture small mammals; G- A meshed trap containing a rat (*Rattusnorvegicus*); H- A plastic rest box containing a woodmouse (*Apodemussylvaticus*) that is released; I- Disinfection of traps (masks and gloves are important to protect humans from zoonotic agents).

Fieldwork: The different steps of the fieldwork are detailed in Fig. 4 and in the ‘sampling description’ section above. All information related to traps and captures is recorded on paper sheets and/or on digital tablets using the KoBo software. These results are described in Suppl. material 1.

Animal dissection: On the day of capture, animals are anaesthetised using isofluorane and euthanised by cervical dislocation, as recommended by Mills et al. (1995). Morphological measures are taken, amongst which were mass and head-body, tail and hind-foot length. Species identification is recorded, based on this morphological information. In case of doubt, only the genus is noted. Sex is recorded and sexual maturity is inferred considering testes length and position (abdominal or descended into the scrotal sac) and seminal vesicle development (visible or not) for males or vaginal opening, nipples (visible or not), pregnancy and uterus size (very thin or thick) for females. Ectoparasites (ticks and fleas) are collected and stored in ethanol at 96°C. Macroparasites that are detected in the cavity are recorded (e.g. cestodes on the liver). This protocol is described in Herbreteau et al. (2011). All information related to individual dissections is recorded on paper sheets and on an MS Еxcel file. Several samples are collected from each animal for further mammal genetics and parasitological (bacteria, viruses, macroparasites, protozoa) analyses. The heart is removed and is stored in PBS at -20°C. The lungs, rectum and a piece of liver are collected and are stored in RNA shield solution (one day at 4° then -20°C). The spleen, the digestive tract, the ears, the left hind-foot and a piece of the tail are collected and are stored in 96% ethanol. Faeces and a piece of lungs are collected and are stored at -20°C. All these samples are given a unique identifier and datamatrix. Metadata associated with the samples and occurrence set were deposited in the CBGP small mammal database (BPM, https://doi.org/10.15454/WWNUPO).

Lastly, all waste products were eliminated using the official incineration process, that is a safe way of destroying hazardous potentially infectious waste, protecting both human and the environment.

**Figure 5.**
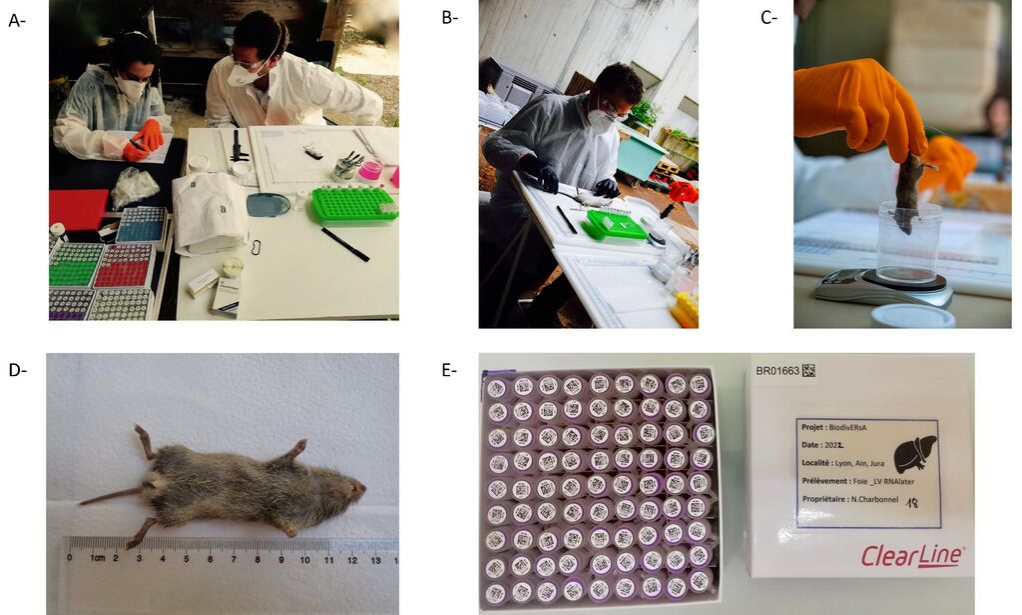
Pictures illustrating the different steps during small mammal dissection. Masks (FFP2), gloves and glasses protect the experimenter who manipulates and dissects the animals. **A**- All tubes are prepared in advance with unique identifier and datamatrix. One colour is dedicated to each type of sample (e.g. red tip for heart in PBS, purple tip for liver in RNA later). Morphological information was recorded on a paper sheet. Dissection instruments are disinfected between each animal. **B**- The experimenter is preparing the animal (here a brown rat *Rattusnorvegicus*) for the dissection. **C**- The experimenter is weighing the animal (here a woodmouse *Apodemussylvaticus)* for the dissection. **D**- A male common vole (*Microtusarvalis*). **E**- Tubes corresponding to liver samples, with unique identifiers and datamatrices, stored in a clearly identified box (unique identifier and datamatrix).

The different steps of the dissection are detailed in Fig. 5.

Ethical statements: Animal capture and handling have been conducted according to the French and European regulations on care and protection of laboratory animals (French Law 2001-486 issued on 6 June 2001 and Directive 2010/63/EU issued on 22 September 2010). The CBGP laboratory has approval (D-34-169-003) from the Departmental Direction of Population Protection (DDPP, Hérault, France) and from the regional ethical committee (Comite d'Ethique pour l'Expérimentation Animale Languedoc Roussillon), for the sampling of rodents and the storage and use of their tissues.

Molecular analyses: DNA was extracted from kidney using Qiagen DNeasy® Blood & Tissue Kit. The specific identification of *Microtus* species and shrews was next performed using CO1 sequencing (BatL5310 and R6036R), following Pagès et al. (2010). The specific identification of *Apodemus* species was determined using DNA fingerprinting (AP-PCR) and the E8S primer, following Bugarski-Stanojević et al. (2013).

## Geographic coverage

### Description

The data were collected in six forested areas in eastern France, within three administrative Departments (Rhône, Ain, Jura).

### Coordinates

44.84 and 48.633 Latitude; 2.021 and 7.734 Longitude.

## Taxonomic coverage

### Taxa included

**Table:** 

Rank	Scientific Name	Common Name
species	*Apodemusflavicollis*	yellow-necked mouse
species	*Apodemussylvaticus*	woodmouse
species	*Crocidurarussula*	greater white toothed shrew
species	*Crociduraleucodon*	bicoloured shrew
species	*Glisglis*	edible dormouse
species	*Microtusagrestis*	short-tailed field vole
species	*Microtussubterraneus*	European pine *vole*
species	*Musmusculus*	house mouse
species	*Myodesglareolus*	bank vole
species	*Neomysfodiens*	Eurasian water shrew
species	*Rattusnorvegicus*	brown rat
species	*Sorexaraneus*	common Eurasian shrew
species	*Sorexcoronatus*	crowned shrew
species	*Sorexminutus*	Eurasian pygmy shrew
species	*Microtusarvalis*	Common vole

## Temporal coverage

### Notes

2020-02-25 through 2022-06-03

## Collection data

### Collection name

CBGP small mammal database (BPM, http://bpm-cbgp.science)

### Collection identifier

https://doi.org/10.15454/WWNUPO

## Usage licence

### Usage licence

Creative Commons Public Domain Waiver (CC-Zero)

### IP rights notes

This work is licensed under a Creative Commons Attribution (CC-BY) 4.0 License.

## Data resources

### Data package title

Small terrestrial mammals (Rodentia, Soricomorpha) along a gradient of forest anthropisation (reserves, managed forests, urban parks) in France.

### Resource link

https://doi.org/10.15468/bn8zz7

### Alternative identifiers

https://www.gbif.org/dataset/688ff587-af92-4f7b-82b5-3ee565afa025

### Number of data sets

1

### Data set 1.

#### Data set name

Occurrence of small terrestrial mammals (Rodentia, Soricomorpha) along a gradient of forest anthropisation (reserves, managed forests, urban parks) in France.

#### Data format

Darwin Core

#### Download URL

https://www.gbif.org/dataset/688ff587-af92-4f7b-82b5-3ee565afa025

#### Data format version

1.5

#### Description

The dataset contains occurrences of small terrestrial mammals (Rodentia and Soricomorpha) trapped in forested areas in eastern France (administrative Departments: Rhône, Ain, Jura) (Charbonnel et al. 2022). The sampling sites correspond to different degrees of anthropisation. Forests included in biological reserves are the less anthropised sites, then public forests and urban parks experience higher levels of anthropisation. Data were collected during spring and autumn 2020 (three to four sampling sites), 2021 (six sampling sites) and 2022 (four sampling sites). These variations in the number of sites between years were due to lockdown restrictions in 2020 or to the legal authorisation to trap around biological reserves granted in 2021 only. The capture of animals was carried out in various types of forests (pine, deciduous, mixed) and in different habitats within urban parks (wooded areas, buildings, hay storage yards, riverside vegetation, restaurants, playground for kids, botanical garden, landfills…). Captures were realised using live traps that were set on the ground for one to 11 nights. During this study period, 1593 small mammals were trapped and identified. They belong to 15 species, amongst which there are nine species of rodents (Muridae, Cricetidae, Gliridae) and six species of shrews (Soricidae). An overview of the captures per species and locality is available in Table 2. Small mammals were weighed (gram) and measured (cm): both body length and tail length. Sexual characteristics were also recorded. This dataset aims to better understand the relationship between small terrestrial mammal biodiversity and health in the context of global change and, in particular, of forest anthropisation. It is part of the European Biodiversa BioRodDis project (https://www6.inrae.fr/biodiversa-bioroddis). Here, we present the data gathered in France. The dataset will enable us to describe the diversity of small terrestrial mammal communities in forested areas corresponding to different levels of anthropisation and to evaluate the variability of this diversity over time, between seasons and between years.

**Data set 1.** 

Column label	Column description
occurrenceID	An identifier for the occurrence (as opposed to a particular digital record of the occurrence). In the absence of a persistent global unique identifier, construct one from a combination of identifiers in the record that will most closely make the occurrenceID globally unique.
scientificName	The full scientific name, with authorship and date information, if known. When forming part of an Identification, this should be the name in the lowest level taxonomic rank that can be determined. This term should not contain identification qualifications, which should instead be supplied in the IdentificationQualifier term.
sex	The sex of the biological individual(s) represented in the Occurrence.
eventDate	The date-time or interval during which an Event occurred. For occurrences, this is the date-time when the event was recorded. Not suitable for a time in a geological context. A variable ("YYYY-MM-DD").
measurementType	The nature of the measurement, fact, characteristic or assertion.
measurementValue	The value of the measurement, fact, characteristic, or assertion.
measurementUnit	The units associated with the measurementValue.
countryCode	The standard code for the country in which the Location occurs.
country	The name of the country or major administrative unit in which the Location occurs.
locationID	An identifier for the set of location information (data associated with dcterms:Location). May be a global unique identifier or an identifier specific to the dataset.
locality	The specific description of the place.
decimalLatitude	The geographic latitude (in decimal degrees, using the spatial reference system given in geodeticDatum) of the geographic centre of a Location. Positive values are north of the Equator, negative values are south of it. Legal values lie between -90 and 90, inclusive.
decimalLongitude	The geographic longitude (in decimal degrees, using the spatial reference system given in geodeticDatum) of the geographic centre of a Location. Positive values are east of the Greenwich Meridian, negative values are west of it. Legal values lie between -180 and 180, inclusive.
recordedBy	A person, group or organisation responsible for recording the original Occurrence.
basisOfRecord	The type of the individual record.
individualCount	Quantity of a species occurrence, for example, the number of individuals.
samplingProtocol	Specify how the "Occurrence" records were obtained.
taxonRank	The taxonomic rank of the supplied scientific name.
kingdom	The full scientific name specifying the kingdom that the occurrence's scientific name is classified under.
phylum	The full scientific name specifying the phylum that the occurrence's scientific name is classified under.
class	The full scientific name specifying the class that the occurrence's scientific name is classified under.
order	The full scientific name specifying the order that the occurrence's scientific name is classified under.
family	The full scientific name specifying the family that the occurrence's scientific name is classified under.
geodeticDatum	The coordinate system and set of reference points upon which the geographic coordinates are based.
coordinateUncertaintyInMetres	The horizontal distance from the given decimalLatitude and decimalLongitude in metres, describing the smallest circle containing the whole of the Location.

## Supplementary Material

A0C1C72C-5B0B-55AC-9149-AE8D9E1F8A33 10.3897/BDJ.10.e95214.suppl1 Supplementary material 1 Detailed information gathered during the field sessions. Data type Trapping results Brief description This Supplementary table details the information gathered during the field sessions. It includes details about the lines and traps set during each field session and for each locality, as well as the results of the trapping detailed for each trap checking. Results can be 'empty open': the trap is open, there is no small mammal in the trap; 'empty closed': for technical issues, the trap is empty and closed, so that it could not have trapped anything; 'not found': the trap is no longer where it has been set (it might have been stolen or moved by large animals, for example). We also provide information on the trap checking, with the preliminary identification of the animal trapped, whether the animal was dead in the trap or released. Note that 67 occurrenceID entries in the occurrence table have no corresponding occurrenceID in this file. This is because these individuals are rats and mice that were trapped by the zoo managers, then frozen and given to us.
Caption : Locality is the specific description of the place; locationID is an identifier for the set of location information (data associated with dcterms:Location); field-sessionID is an identifier for the session of trapping; Trap-settingDate is the date during which traps were set ("YYYY-MM-DD"); Trap-lineID is an identifier for the trap lines; Trap-checkingDate is the date when traps were checked (; Trap-checking-number is the number of a particular trap checking for a given field-sessionID; TrapID is an identifier for the traps, for a given locality and a given field-sessionID; decimalLatitude is the geographic latitude (in decimal degrees, using the spatial reference system given in geodeticDatum) of the trap; decimalLongitude is the geographic longitude (in decimal degrees, using the spatial reference system given in geodeticDatum) of the trap; geodeticDatum is the coordinate system and set of reference points upon which the geographic coordinates are based; coordinateUncertaintyInMetres is the horizontal distance from the given decimalLatitude and decimalLongitude in metres, describing the smallest circle containing the whole of the Location; trapping-result is the result of a given trap checking; FieldObservation includes comments relative to the trap checked (species trapped, individual found alive, dead in the trap, animal released or not…); dissectionDate is the date during which the animal were dissected ("YYYY-MM-DD"); occurrenceID is an identifier for the Occurrence (as opposed to a particular digital record of the occurrence). In the absence of a persistent global unique identifier, construct one from a combination of identifiers in the record that will most closely make the occurrenceID globally unique. This is the same occurrenceID as in the dataset published here and in GBIF. File: oo_747366.csv https://binary.pensoft.net/file/747366 Julien Pradel, Marie Bouilloud, Anne Loiseau, Sylvain Piry, Maxime Galan, Emmanuelle Artige, Guillaume Castel, Julien Ferrero, Romain Gallet, Geoffrey Thuel, Nathalie Vieira, Nathalie Charbonnel

ECCC018B-FA58-5B2F-9497-6CD69CEEE079 10.3897/BDJ.10.e95214.suppl2 Supplementary material 2 AP-PCR protocol adapted from Bugarski-Stanojevic et al. (2013) for molecular identification of *Apodemus* species Data type Protocol Brief description AP-PCR protocol adapted from Bugarski-Stanojevic et al. (2013) for molecular identification of *Apodemus* species. File: oo_746059.pdf https://binary.pensoft.net/file/746059 Loiseau, A.

## Figures and Tables

**Table 1.** List of sampling sites including locality (and locationID), coordinates (latitude and longitude of the centroid of the area covered by the traps), the trapping line ID and the landscape type. The number of small mammals trapped and dissected for each site and session (spring 2020; autumn 2020; spring 2021; autumn 2021; spring 2022) is provided.

**Locality**	**LocationID**	**Centroid_x**	**Centroid_y**	**Trapping_LineID**	**Landscape_type**	**Number_dissection_session**
Lyon, Parc de la Tête d’Or	FRPLTO	4.85554195	45.77817576	ASI, CT, CYN, EMB, FAUV, GIF, ILE, IVEL, JDP, LAM, LUZ, MAM, MOM, OBS, PM, PON, PRI, SBO, SBP, SEV, VAC, VOL	Urban park	84;94;82;124;85
Marcy l'etoile, Domaine Lacroix Laval	FRPDLL	4.72146918	45.78982081	A, Abis, B, C, CREP, D, E, F, G, H, I, PAI, SEL	Urban park	0;41;96;93;11
Cormaranche en Bugey	FRFCOR	5.62549550	45.93477080	A, AA, AB, AC, AD, B, C, D, E, F, G, H, I, J, K, Kbis, L, M	Managed forest	29;108;94;95;8
Arviere, La Griffe au diable	FRFGRI	5.75865886	45.93370736	S, T, V, W, X, Y, Z	Protected forest	0;0;95;41;0
Mignovillard	FRFMIG	6.16343853	46.76358715	A, AA, AC, AD, AG, B, BB, C, CC, D, DD, E, F, G, H, I, J, K, L, M, N, P, Q	Managed forest	41;81;69;98;25
Esserval-Tartre, La Glacière	FRFGLA	6.02525045	46.84288358	S, T, V, W, X, Y, Z	Protected forest	0;0;33;66;0

**Table 2.** Occurrence (number of individuals) of the small mammal species trapped in the different localities surveyed. FRPLTO: Lyon, Parc de la Tête d’Or; FRPDLL: Marcy l'étoile, Domaine Lacroix Laval; FRFCOR: Cormaranche en Bugey; FRFGRI: Arvière, La Griffe au diable; FRFMIG: Mignovillard; FRFGLA: Esserval-Tartre, La Glacière. The total number of individuals trapped is also indicated for each locality and for each species.

**Small mammal species**			**LocalityID**				**Total number of individuals**
	**FRPLTO**	**FRPDLL**	**FRFCOR**	**FRFGRI**	**FRFMIG**	**FRFGLA**	
*Apodemus* sp.	0	0	0	0	1	0	1
Soricomorpha	0	1	0	0	1	0	2
*Apodemusflavicollis*	0	38	118	51	89	33	329
*Apodemussylvaticus*	177	108	55	17	43	11	411
*Crociduraleucodon*	0	0	13	0	17	0	30
*Crocidurarussula*	83	15	0	0	0	0	98
*Glisglis*	0	0	8	0	9	0	17
*Microtusagrestis*	0	0	2	1	1	0	4
*Microtusarvalis*	29	1	0	1	0	0	31
*Microtussubterraneus*	0	0	0	1	3	0	4
*Musmusculus*	89	1	0	0	0	0	90
*Myodesglareolus*	0	77	134	61	138	52	462
*Neomysfodiens*	0	0	2	0	0	1	3
*Rattusnorvegicus*	91	0	0	0	0	0	91
*Sorexaraneus*	0	0	0	0	3	0	3
*Sorexcoronatus*	0	0	2	4	7	2	15
*Sorexminutus*	0	0	0	0	2	0	2
Total number of individuals	469	241	334	136	314	99	1593
Total number of species detected	5	6	8	7	10	5	15
