# Supplementary material for: Small terrestrial mammals (Rodentia and Soricomorpha) along a gradient of forest anthropisation (reserves, managed forests, urban parks) in France
Source: Biodivers Data J. 2022 Dec 30;10:e95214. doi: 10.3897/BDJ.10.e95214 (PMC9836417; doi:10.3897/BDJ.10.e95214)
Supplement: Supplementary material 2 — AP-PCR protocol adapted from Bugarski-Stanojevic et al. (2013) for molecular identification of Apodemus species [file bdj-10-e95214-s002.pdf]

AP-PCR protocol adapted from Bugarski-Stanojevic et al. 2013 for molecular identification of *Apodemus* species

By Loiseau, A. 2022-09-13

DNA extraction
Total DNA was extracted from kidney in 96° ethanol using the BioBasics kit and the associated recommendations, or Chelex extraction method.

AP-PCR

The isolated DNA was amplified by AP-PCR using the E8S primer (exone 8) 5′-TAAATGGGACAGGTAGGACC-3′ (Metabion). PCR reaction was performed using 3 µl of ES8 primer, 2 µl of Qiagen multiplex PCR kit containing hotstar Taq DNA polymerase, and 2 µl of DNA.

The temperature profile was: 95 °C for 30 sec, 47 °C for 1.30 min; and 72 °C for 1 min. This was repeated during 45 cycles.

PCR amplifications were analyzed using agarose gel (1.5 % and 40 min of migration). The profiles obtained for *Apodemus sylvaticus* (SYL) and *Apodemus flavicollis* (FLA) are shown below.

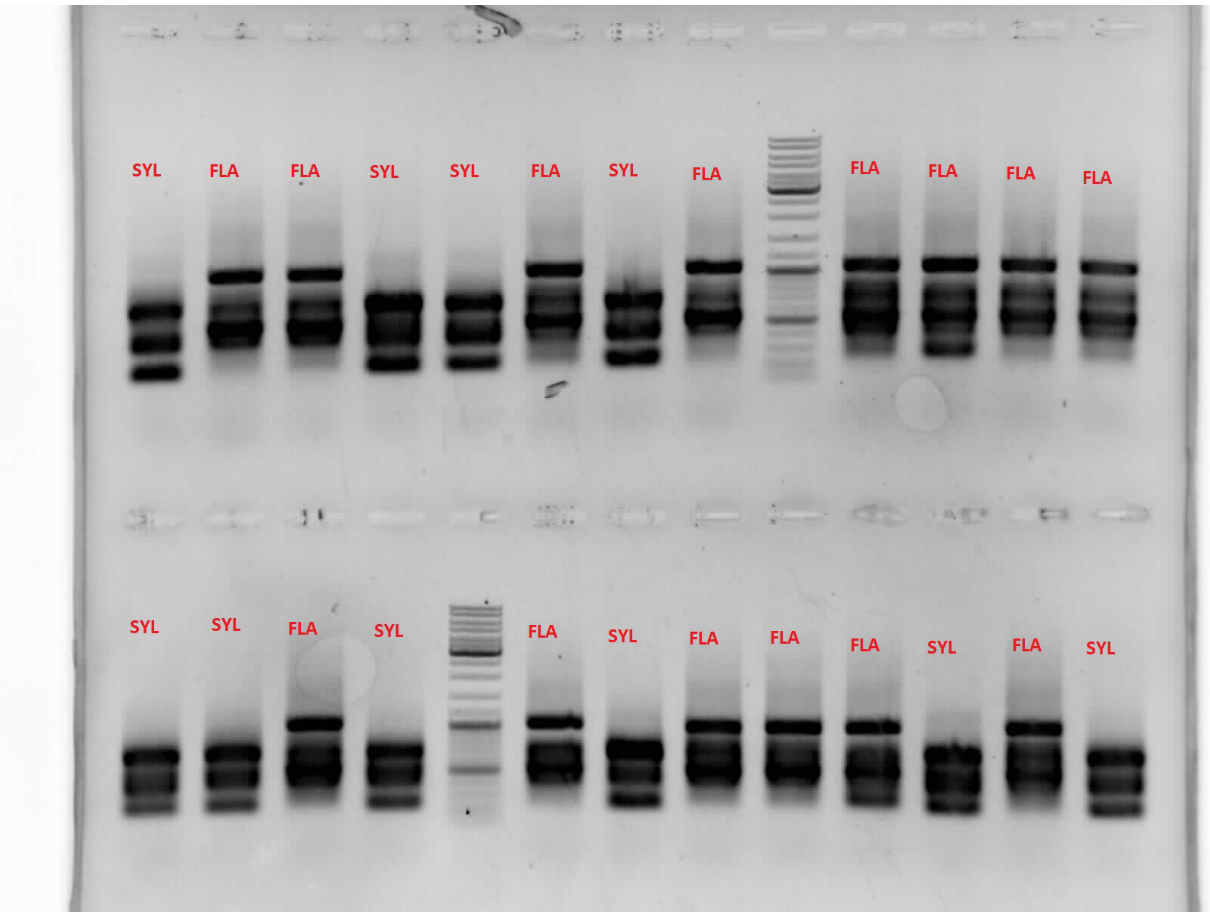

Reference

Bugarski-Stanojević V, Blagojević J, Adnađević T, Jovanović V, Vujošević M (2013) Identification of the sibling species *Apodemus sylvaticus* and *Apodemus flavicollis* (Rodentia, Muridae)—Comparison of molecular methods. Zoologischer Anzeiger – A Journal of Comparative Zoology 252 (4): 579-587. https://doi.org/10.1016/j.jcz.2012.11.004
